# Comparison of differentiation potential of male mouse adipose tissue and bone marrow derived-mesenchymal stem cells into germ cells

**Published:** 2013-12

**Authors:** Maryam Hosseinzadeh Shirzeily, Parichehr Pasbakhsh, Fardin Amidi, Kobra Mehrannia, Aligholi Sobhani

**Affiliations:** *Department of Anatomy, Faculty of Medicine, Tehran University of Medical Sciences, Tehran, Iran.*

**Keywords:** *Mesenchymal stem cells*, *Infertility*, *Germ cells*, *Retinoic acid*

## Abstract

**Background:** Recent publications about differentiation of stem cells to germ cells have motivated researchers to make new approaches to infertility. In vitro production of germ cells improves understanding differentiation process of male and female germ cells. Due to the problem of using embryonic stem cells (ESC), it’s necessary the mentioned cells be replaced with some adult multi-potent stem cells in laboratories.

**Objective: **The aim of this study was to obtain germ cells from appropriate source beyond ESC and compare differential potentials of adipocytes derived stem cells (ADMSCs) with bone marrow derived stem cells (BMMSCs).

**Materials and Methods:** To find multi-potential entity, after providing purified ADMSCs and BMMSCs, differentiation to osteoblast and adipocyte was confirmed by using appropriate culture medium. To confirm mesenchymal lineage production superficial markers (expression of CD90 and CD44 and non-expression of CD45 and CD31) were investigated by flowcytometry. Then the cells were differentiated to germ cells in inductive medium containing retinoic acid for 7days. To evaluate germ cells characteristic markers [Dazl (Deleted in azoospermia-like), Mvh (Mouse vasa homolog gene), Stra8 (Stimulated by retinoic acid) and Scp3 (Synaptonemal complex protein 3)] flowcytometry, imunoflorescence and real time PCR were used.

**Results:** Both types of cells were able to differentiate into osteoblast and adipocyte cells and presentation of stem cell superficial markers (CD90, CD44) and absence of endothelial and blood cell markers (CD31, CD45) were confirmative The flowcytometry, imunoflorescence and real time PCR results showed remarkable expression of germ cells characteristic markers (Mvh, Dazl, Stra8, and Scp3).

**Conclusion: **It was found that although ADMSCs were attained easier and also cultured and differentiated rapidly, germ cell markers were expressed in BMMSCs significantly more than ADMSCs.

This article extracted from M.Sc. thesis. (Maryam Hosseinzadeh Shirzeily)

## Introduction

One of the most common problems between couples is infertility. Infertility has been defined as inability to conceive a child after one year of regular sexual intercourse without contraception method (1). To dissolve infertility problems, lots of treatment strategies such as hormone therapy, in vitro fertilization (IVF) and intra cytoplasmic sperm injection (ICSI) have been studied (2). The mentioned methods were not instrumental in some couples that motivated researchers to provide new hypotheses. Stem cells attracted much attention due to their noticeable potentials. Recently, stem cells have been used extensively for the purpose of fertility treatment and in vitro production of gametes (3-6).

In the last decades, the in vitro experiences showed that the germ cells can be retrieved not only from Embryonic Stem Cells (ESCs) but also from adult bone marrow stem cells and even from skin tissue (3-11). Hubner *et al* in 2003 succeeded in differentiating mouse ESCs into oocytes like cells, for the first time (3) .This successful experiments were repeated in other studies (4-7). Because using ESC has been associated with tumorogenesis and also ethical criticisms, Researchers were suggested, ESC to be replaced with some adult stem cells (12-14).

In this way scientists focused on use of adult stem cells derived from bone marrow stem cells which have presented some new potential that have motivated lots of biological studies beyond traditional approaches. Nayernia *et al* in 2006 induced differentiation of mouse Bone Marrow Mesenchymal Stem Cells (BMMSCs) to male gametes in culture medium contained Retinoic Acid (RA), for the first time (8). 

In following studies, Drusheimer *et al* in 2007 and Hau *et al* in 2009, succeeded to attain male gametes from human BMMSCs by using RA in laboratory    (9,  10). Over the time, using BMMSCs have been gone to be replaced by other stem cells due to a highly invasive method to extract the cells, morbidity, and low cell number in aspiration (15). Since the adipose tissue is more accessible, many researchers were suggested that stem cells from mentioned sources can be replaced by Adipocytes Mesenchymal Stem Cells (ADMSCs) due to easy extraction within less painful process (16, 17). 

In following studies, researchers were used other sources of adult stem cells to differentiate into germ cells. Song *et al* in 2011 succeeded to attain putative oocyte-like cells from porcine adipose, skin and ovarian tissues in medium containing follicular fluid (18). To obtain differentiated lineages from stem cells, genetic manipulations of transcription factors or signaling molecules have been found effective (, 20). One of the signaling molecules was Retinoic Acid (RA). RA is the biologically active form of vitamin A and widely associated with in growth and differentiation of variety of cell types (21). It acts by binding to nuclear RA receptor (22).

The mentioned substance precipitates cells entry into meiotic phase in genital crest located in the central region of the mesonephros and in vitro causes induction of stem cell differentiation (22, 23). Some articles claimed that this extrinsic inductive factor plays an important role in embryonic stem cells replication and differentiation to sexual progenitor cells (23). Comparing differentiation capability of BMMSCs and ADMSCs can provide more accessible resources of stem cells if ADMSCs could activate as well as other previously used stem cells. The present study was designed to compare differentiation capability of BMMSCs and ADMSCs to germ cells. Also the efficacies of RA as essential supplements for culture medium were assessed.

## Materials and methods

**Isolation and culture the cells**

In this experimental study, all experiments which were conducted on animals were in accordance with the guidance of ethical standards for research on laboratory animals of Tehran Medical University. To achieve BMMSCs, the femurs and tibias bones from 4-8 week old male NMRI mice were aspirated and cultured regarding to Jamous *et al* experience (24). Adipose tissue was surgically attained from the fatty tissue surrounding the testis of 6-8 week-old NMRI mice and sliced into small pieces and incubated in 2% collagenase solution in incubator at 37^o^C for an hour. Then, DMEM (Invitrogen, USA) with FBS 10% (Invitrogen, USA) was added to neutralize collagenase.

The suspensions of both lineages of cells were plated in 15 mL tubes and centrifuged at 1500 RPM for 5 min to pellet cell debris. The cells were maintained in a culture flasks containing Dulbecco’s Modiﬁed Eagle's Medium (DMEM) (Invitrogen, USA) with Fetal Bovine Serum (FBS) 10% (Invitrogen, USA), Penicillin 10 IU/ml and Streptomycin 10 IU µg/ml (Invitrogen, USA). The cells were incubated at 37^o^C in a humidified atmosphere of 95% air and 5% CO_2_. The medium was changed within the next day. To detach non adherent cells, the culture media were changed every 3 days and passaged after 2 weeks when the cells reached about 90-95% on fluence, by using trypsin (Invitrogen, USA). 

**Induction of osteogenic and adipogenic differentiation**

BMMSCs and ADMSCs were cultured as mentioned above. After reaching 80-90% confluence (fourth passage), to induce osteogenic and adipogenic lineage, the cells were cultured in pre-modified osteogenic and adipogenic media (Bonyakhte Company, Rasht, Iran). Adipogenic medium contained DMEM/F12 with 10% FBS, dexamethasone 1µM (SIGMA, USA), 500 µM IBMX (SIGMA, USA), indomethacin 60µM (SIGMA, USA) and insulin 5µM (SIGMA, USA). 

Also osteogenic medium contained DMEM/F12 with 10% FBS, ascorbate-2-phosphate 50µM (SIGMA, USA), and dexamethasone 0.1µM (SIGMA, USA) (26). The conditioned media were incubated in a 95% humidified, 5% CO_2_ atmosphere at 37^o^C. Also they were changed with a fresh one every 3-4 days. The mineralized and calcified spots in osteogenic media were stained with Alizarin Red Solution (SIGMA, USA). 

The cells were washed twice with PBS (Invitrogen, USA) and fixed with formaldehyde 4% for 20min. prior to staining, the cells were washed trice with PBS (Invitrogen, USA) and then 500 µl of Alizarin Red Solution (SIGMA, USA) was added for 10 minutes. To see the cells by light microscope, the cells were rinsed off. The red spots were representative for differentiation to osteoblasts. To stain adipogenic media, the steps were similar to those mentioned above except adding 500µl of Oil Red O (Merck, Germany) instead of Alizarin Red Solution. 

**Analysis of Cell Surface** **Markers**

In order to prove the existence of mesenchymal stem cells achieved from BMMSCs and ADMSCs, superficial markers were analyzed using flowcytometry (Becton Dickinson FACS Calibur flow cytometer) according to Chemicon protocol (18, 26). The cells were cultured then after fourth passage they were harvested with tripsin (Invitrogen, USA) and finally, 10^5^-10^6^ cells were used for analysis. Flowcytometric assays (Becton Dickenson, Franklin Lakes, NJ) were performed and Win MDI 2.9 software was used for analysis.

To identify mesenchymal stem cells CD markers were investigated. Meanwhile flowcytometry analysis, CD90 (11-0900, eBioscience, UK) and CD44 (ab25064, abcam, USA) were representative for mesenchymal stem cells CD45 (ab25670, abcam, USA) and CD31 (ab95652, abcam, USA) were expressed by hematopoietic and endothelial stem cells respectively. To evaluate the expression, cells were incubated in 5 mg/mL FITC conjugated. FITC mouse IgG2a Isotype control (11-4724, eBioscience, UK), Rat IgG1 isotype control (ab18412, abcam, USA) were used as isotype control.

**Induction of** **BMMSCs and ADMSCs to germ cells**

To culture the cells, they were incubated in DMEM (Invitrogen, USA) with FBS 10% (Invitrogen, USA), penicillin/streptomycin (Invitrogen, USA) and retinoic acid 10^-5^µM (Invitrogen, USA) was added, at 37^o^C in a humidified atmosphere of 95% air and 5% CO_2_ for 7 days. DMEM was changed every 2 days. In the control group, the ADMSCs and also BMMSCs were cultured in DMEM without differentiation-inducing factors for 7days (control groups). The achieved differentiated cells were assessed for germ cell identifying characteristics. By using a combination of immunofluorescence, flow cytometry and real time PCR, the cells differentiated to germ cells were distinguished from the control group. 

**Immunofluorescence**

Immunofluorescence was performed to assess differentiation of BMMSCs and ADMSCs to germ cells. The technique was started following stem cells induction to evaluate lack of germ-cell marker expression in those cells cultured in differentiation/growth factors deprived media. 10^5^-10^6 ^cells were washed with cold PBS (Invitrogen, USA) and then fixed with formaldehyde 4% for 20 min. To make the cells permeable, Triton 100-X (Invitrogen, USA) was used for 15 min. Non-specific proteins were blocked by using (1% bovine serum albumin in phosphate-buffer sulin pbs/tween) at room temperature for 45 min. 

The diluted primary antibody combination, [Dazl (sc-27333, SANTA CRUZ 1:50) and Mvh (701365, antibodies-online 1:50)], was added to cells and the product was preserved at 4^o^C for a night. Following washing the cells thrice by PBS (spending 3 min for each time), secondary antibodies [Rat anti-Rabbit IgG (30087, antibodies-online, 1:100) and donkey anti-goat IgG (sc-2024, SANTA CRUZ, 1:100)] were added. The cells were incubated at room temperature for 2 hours. To get rid of additional antibodies, the cells were washed out twice with Phosphate Buffer Saline (PBS) (spending 3 min for each time). To evaluate the expression, cells were incubated in 5 mg/mL FITC conjugated. 

FITC mouse IgG1 Isotype control (11-4714, eBioscience, UK, 1:200), Rabbit polyclonal IgG (ab27472, abcam, USA, 1:200) were used as isotype control. With the purpose of staining nucleus, DAPI was used. In a dark room, 2 drops of the solution were distilled to each box and then extracted after 15 seconds to be washed with PBS (Invitrogen, USA). By using florescent microscope, brilliant green was representative for the cells that presented the target proteins (18).

**Flowcytometry**

Following differentiation induction, flowcytometry was performed to evaluate differentiation of BMMSCs and ADMSCs to germ cells by identifying expression of Mvh and Dazl proteins according to Chemicon protocol. After inducing differentiation to the cultured cells, they were rinsed off with tripsin (Invitrogen, USA) and harvested. Finally, 10^5^-10^6^ cells were fixed in 1 ml of paraformaldehyde 4% (Sigma, USA) at 4o^C^ for 30 min. The packs were centrifuged with speed 1500RPM for 5 min. The cells were incubated with Bsa 5% at room temperature for 8 minutes. 

Then, initial antibodies [Dazl (SANTA CRUZ, sc-27333, 1:50) and Mvh (antibodies-online, 701365, 1:50)] were added following PBS washing (Invitrogen, USA) and adding Bsa 5% for 8 minutes. The cells were incubated in the combined solution for a night. Within the next day, the cells were washed with PBS and centrifuged with speed of 1500 RPM. The cells were placed in our prepared secondary antibody solution [FITC diluted ideally: Rat anti-Rabbit IgG (antibodies-online, 30087, 1:100) donkey anti-goat IgG (SANTA CRUZ, sc-2024, 1:100)] at 4^o^C for 30-45 min. The cells were washed twice by PBS and centrifuged with speed of 1500RPM for 5 min. Prior to flow cytometry (Becton Dickinson FACS Calibur flow cytometer), confluent cells were fixed with 10% paraformaldehyde at room temperature. To evaluate the expression, cells were incubated in 5 mg/mL FITC conjugated. FITC mouse IgG1 Isotype control (11-4714, eBioscience, UK, 1:200), Rabbit polyclonal IgG (ab27472, abcam, USA, 1:200) were used as isotype control. Flow cytometric assays (Becton Dickenson, Franklin Lakes, NJ) were performed and WinMDI 2.9 software was used for analysis.

**Real time PCR**

To assess the expression of Deleted in azoospermia-like (Dazl), Mouse vasa homolog gene (Mvh), Stimulated by retinoic acid (Stra8) and Synaptonemal complex protein 3 (Scp3) after differentiation, real time PCR was performed. To achieve RNAs from cells, RNX-Plus (Sinagene, Tehran, Iran) was used while DNAs Ι (Fermenta, Canada) adding was performed to get rid of any DNAs in the residual culture flask. Complementary DNA (cDNA) was attained from extracted RNAs regarding “BioRT cDNA First Strand Synthesis Kit “protocol. The procedure was followed by carrying out real time PCR. A prepared master mix (Amplicon) was transferred to a specific tube. To accomplish full genome sequence, the specimens were evaluated by Illumina device. 

The primer sequences for the genes are illustrated in Table I. The Eco real time PCR software was modified: enzyme activation 95^o^C for 15 minutes, denaturation 95^o^C for 10 seconds, annealing 56^o^C for 20 seconds, synthesis 72^o^C. After 35 cycling, the specimens were plated in agarose gell 2% electrophoresis followed by cyber staining. To assess gene expression quantity, we calculated the normality by using 2^-ΔΔCt^ formula while GAPDH was used as housekeeping. 

**Statistical analysis**

In present study we used t-test pair. P-value less than 0.05 were considered as significant. SPSS software version 16.0 was used to analyze the data. 

**Table I:** Primer used for Real time PCR

Genes	Primer sequences	Amplicon length	Gene Bank code
Mvh	F:-5’ CGAAACATAGGTGATGAAAGAAC	193bp	NM_001145885
	R:-5’ CCACTGAAGTAGCAACAAGAAC		
Dazl	F:-5’ ATCAGCAACCACAAGTCAAG	188bp	NM_001145885
	R:-5’ CAAATCCATAGCCCTTCG		
Scp3	F:-5’ AAAGCATTCTGGGAAATCTG	188bp	NM_011517
	R:-5’ GTACTTCACCTCCAACATCTTC		
Stra8	F:-5’ GGCAGTTTACTCCCAGTCTG	166bp	NM_009292
	R:-5’ TTCCTTGACCTCCTCTAAGC		
GAPGH	F:5’ -AACTTTGGCATTGTGGAAGG-3’	132bp	NM_008084
	R: 5’ –GGATGCAGGGATGATGTTCT-3’		

## Results

**Figure 1:**
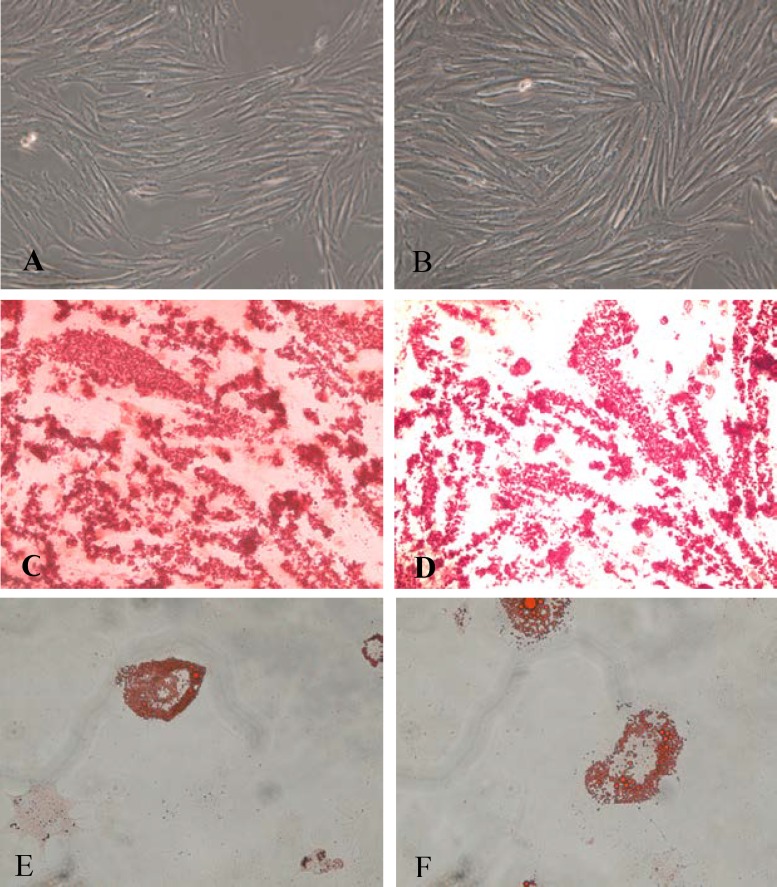
Bone marrow mesenchymal stem cells (A) and adipose tissue mesenchymal stem cells (B) prior to differentiation: fusiform and fibroblastic like cells. Differentiation to Ostesoblast: Bone marrow mesenchymal stem cells (C) and adipose tissue mesenchymal stem cells (D), Alizarin Red staininig. Differentiation to adypocytes: Bone marrow mesenchymal stem cells (E) and adipose tissue mesenchymal stem cells (F), Oil Red-O staininig. [X200 magnification].

Our results showed that spindle shaped BMMSCs and ADMSCs replication reached as much as 80-90% of the original concentration within 5 and 8 days respectively (Figure 1 A and B). To evaluate differentiation to osteoblast all the cells (ADMSCs and BMMSCs) stained with Alizarin Red S and the red spots were representative for differentiation to osteoblasts (Figure 1 C and D). Also to evaluate differentiation to adipocytes, the cells stained with Oil-red O and accumulation of lipid droplets in the cells were clear (Figure 1 E and F). 

**Figure 2:**
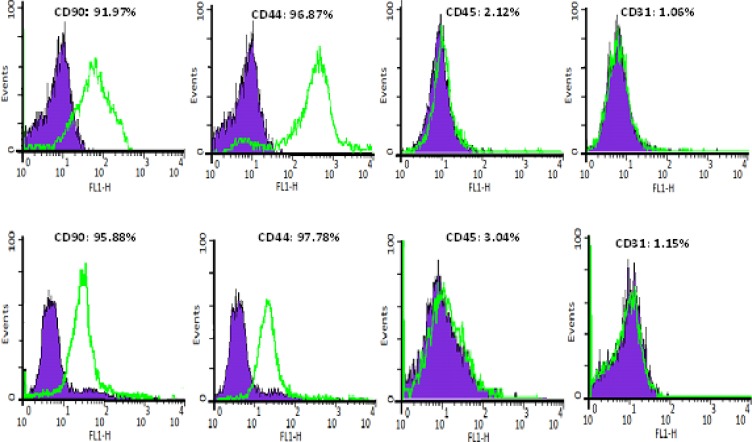
Flow cytometry analysis results: expression of superficial markers in bone marrow mesenchymal stem cells (upper) and adipose tissue mesenchymal stem cells (lower). Expression of superficial characteristics of stem cells (CD44 and CD90) and non-expression of hematopoietic and endothelial markers are illustrated (CD45 and CD31).

To identify bone marrow and adipose tissue stem cells, flowcytometry was performed. The results revealed remarkable expression of stem cell markers specially CD44 and CD90. Subsequently there were no evidences for CD31 and CD45 expression. Regarding to flowcytometry findings, the entity of mesenchymal stem cells was confirmed (Figure 2). To evaluate the induction of differentiation, the results were presented in 2 groups, case and control. 

**Figure 3:**
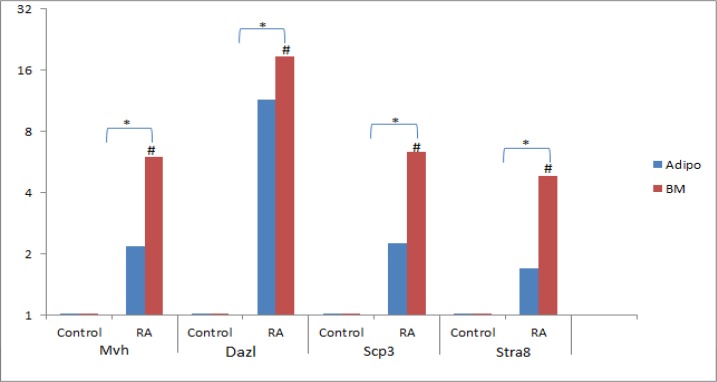
*: Significat p-value(<0.05).

**Figure 4:**
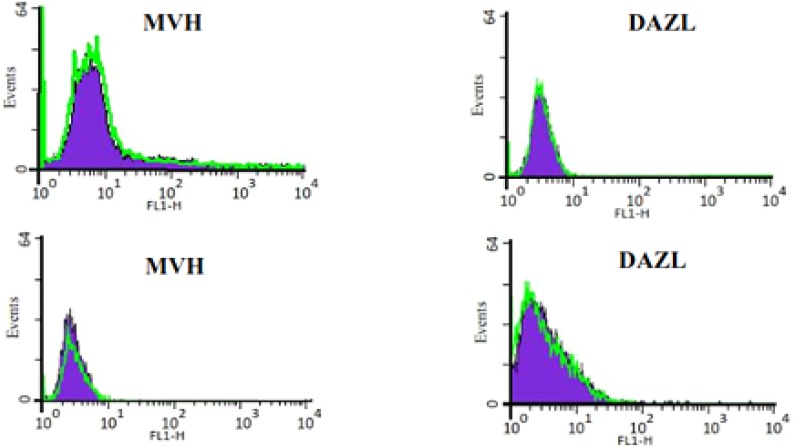
Flow cytometry results for bone marrow mesenchymal stem cells (upper) and adipose tissue mesenchymal stem cells (lower) in a medium without differentiating factor for 7 days following the fourth passage. The cells did not express germ cell markers (Dazl and Mvh).

**Figure 5:**
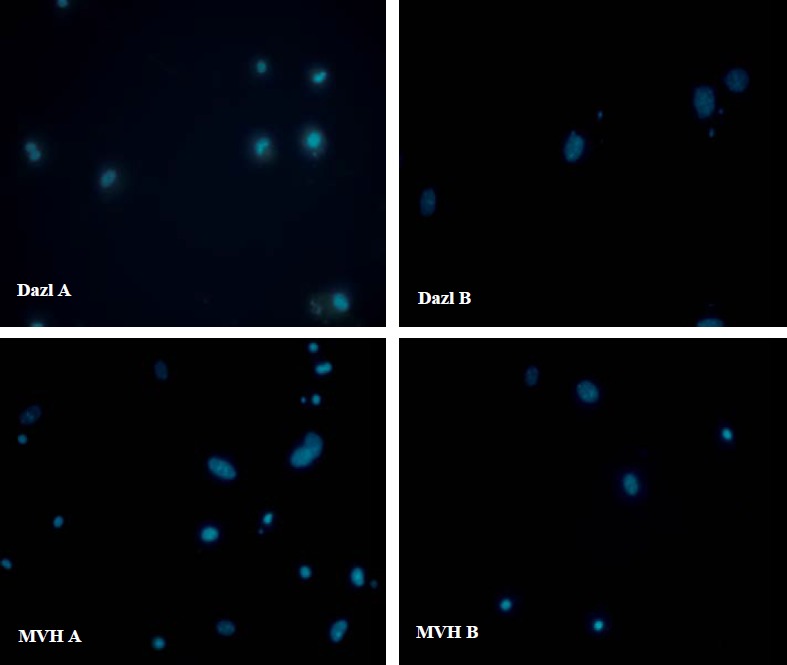
Imunoflorescence staining of Mvh and Dazl (green) in bone marrow mesenchymal stem cells (a) and adipose tissue mesenchymal stem cells (b) [the group cutured in a medium without differentiating factor]. The nucleus were stained with DAPI (blue). Both kinds of the cells did not express Dazl and Mvh. [X400 magnification] .

**Figure 6:**
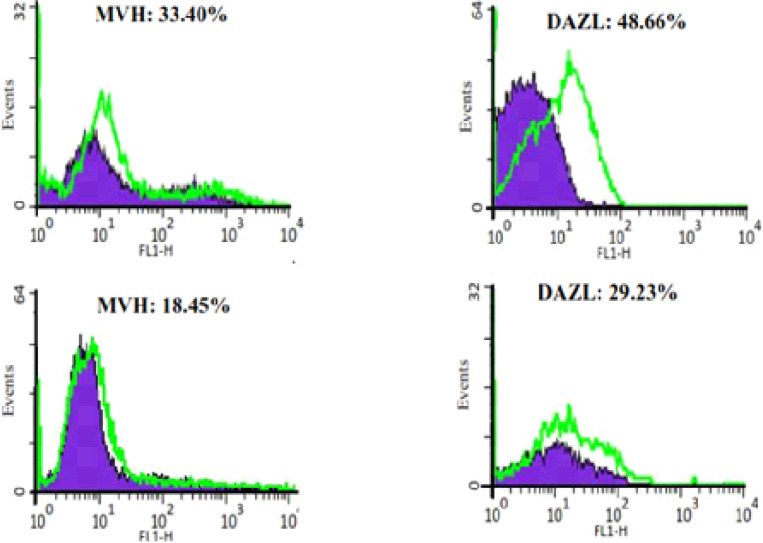
Flow cytometry results for bone marrow mesenchymal stem cells (upper) and adipose tissue mesenchymal stem cells (lower) after differentiating to germ cells with RA for 7 days. The cells expressed germ cell markers (Dazl and Mvh).

**Figure 7:**
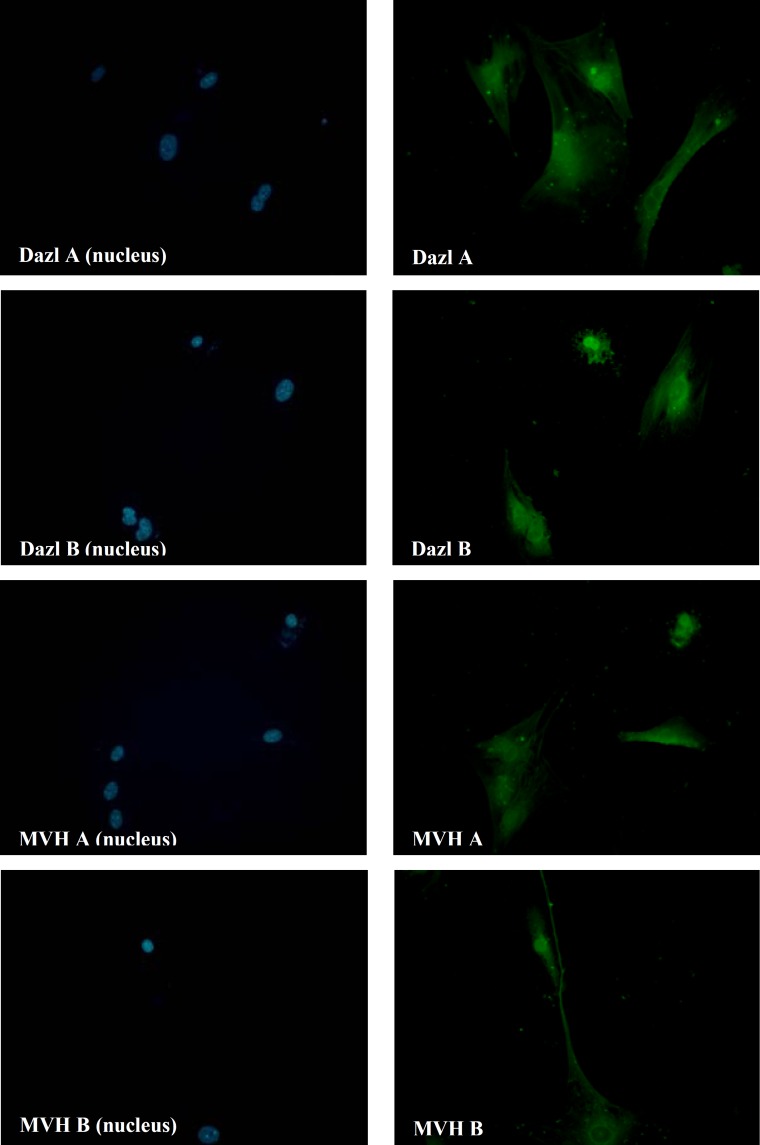
Imunoflorescence staining of Mvh and Dazl (green) in germ cells derived from bone marrow mesenchymal stem cells (a) and adipose tissue mesenchymal stem cells (b). The nucleus was stained with DAPI (blue) [the group cultured in RA-enriched medium]. Both kinds of the cells differentaited to germ cells and expressed Dazl and Mvh. [X400 magnification].

In control group, ADMSCs and BMMSCs, cultured in DMEM without differentiation-inducing factors for 7 days, Q-PCR was performed. The results revealed no expression of Dazl, Mvh, Stra8 and Scp3 (Figure 3). Also performance of flowcytometry and Immunofluorescence revealed no expression of germ cells characteristics (Dazl or Mvh) (Figure 4 and 5). In the other group, ADMSCs and also BMMSCs cultured in a conditioned medium enriched with retinoic acid for 7 days, Rreal time PCR results revealed remarkable expression of Dazl, Mvh, Stra8 and Scp3. According to this experience, the quantity of expressed genes in BMMSCs was significantly more than ADMSCs (Figure 3). Also flowcytometry and immunofluorescence showed expression of Dazl and Mvh in BMMSCs and ADMSCs, in flowcytometry the quantity of expressed in BMMSCs was more than ADMSCs (Figure 6 and 7).

Adipose tissue mesenchymal stem cells

Bone marrow mesenchymal stem cells

## Discussion

Recently, multipotential undifferentiated stem cells have been studied by lots of researchers to provide new remedy for infertility (25). Previous studies have showed that some cells differentiated from embryonic or adult stem cells could express germ cells specific markers (3-11, , 26). In the present study, to attain mesenchymal stem cells, cells were extracted from bone marrow and adipose tissue. Both of them have been previously used for treatment of other diseases due to their noticeable replication potential and also the capability of differentiation to endothelial, epithelial, muscular, hepatical and neural cells (27-29). 

In this study, spindle shaped fibroblast-like cells were attained from differentiation of BMMSCs and ADMSCs following consecutive culture medium changing. Also this experience showed that sequential culture medium changing could impede adherence of non-mesenchymal and blood cells to culture plates. Moreover, it was seen that superficial antigens CD45 and CD31 were not expressed in the cultured cells, although CD44 and CD90 were expressed noticeably. These findings were similar to a study conducted by Dominici *et al* in 2006 (28). 

To prove the multi-potential entity of BMMSCs and ADMSCs, differentiation was induced by adipocyte and osteoblast conditioned medium and illustrated by Oil-red O and Alizarin red S staining for adipocytes and osteoblasts respectively. Overall, lack of expression of superficial characteristics of blood and endothelial cells, remarkable expression of mesenchymal markers, the presence of spindle shaped morphology of cells, considerable potential for replication and differentiation especially to mesenchymal progenitors (adiposecyte and osteoblast) confirmed the essence of studied cells that were naturally mesenchymal stem cells (18, 26). 

Although bone marrow mesenchymal stem cells are the hopeful source but it is an invasive procedure to obtain them. This method is invasive for human due to the need of big bones with general or spinal anesthesia for aspiration which is harmful for patient (16, 26). From both experimental and theoretical points of view, comparing ADMSCs and BMMSCs, adipose tissue cells were extracted more easily within less painful procedure in a relatively larger number of cells and rapid replication (29, 30). Also In the present study very large period of time was spent to grow and proliferation of BMMSCs.

In the next step, ADMSCs and BMMSCs were cultured in a medium containing RA for 7 days. Following that, to evaluate differentiation potential to germ cells, immunofluorescence, flowcytometry and real time PCR were used. To detect differentiated cells, Dazl (Deleted in azoospermia-like), Mvh (Mouse vasa homolog gene), Stra8 (Stimulated by retinoic acid) and Scp3 (Synaptonemal complex protein 3) were investigated as specific superficial markers for germ cells. DAZL is the prenatal and postnatal markers in males and females germ cells (31). Scp3 is a major structural component of the meiosis synaptonemal complex in spermatogenesis and oogenesis (32). Stra8 is the regulation of meiotic initiation in both male and female (33).

Among them, Mvh was the only reliable marker to find germ cell lineage. This gene expresses from post migration stages to post meiosis stages and has role in proliferation and differentiation of primary germ cells (31). Mvh is also known as probable ATP-dependent RNA helicase witch is a general marker for all germ cells that has not been found to be expressed by other kind of cells (34, 35). In our study, a group of cells were cultured and studied as control group. Stem cells in this group was studied without receiving any kinds of differentiation factors.

As it was expected, the cells in this group did not express superficial markers, neither in gene (Mvh, Dazl, Stra8, and Scp3) nor proteins (Dazl, Mvh) level. Previous studies have been showed that the same results (9-11, 26). Nevertheless, some studies have reported spontaneous differentiation of ESCs into germ cells (34, 36). In the other group ADMSCs and BMMSCs were cultured in a medium enriched with retinoic acid for 7 days. Differentiation to germ cells in this group resulted in remarkable expression of Mvh, Dazl, Stra8 and Scp3. Also, it was found that the expressed markers in germ cells derived from BMMSCs were significantly more than those in ADMSCs. 

In the present study, mentioned results were confirmed by immunofluorescence staining and flowcytometric analysis for Mvh and Dazl and real time PCR for Mvh, Dazl, Stra8, and Scp3. To the best of our knowledge, production of germ-like cells derived from ADMSCs has not been previously reported. By the present study, germ-like cells were differentiated from mouse adipose tissue in a medium enriched with RA. RA has been described primarily as a cell cycle modifier to make cells enter to meiosis (22). 

In previous experiences, cellular mitosis division and subsequent meiosis were precipitated in vitro by using RA (8, 9, 36, 37). As it was expected, RA has played an important role in replication and differentiation of BMMSCs and ADMSCs to germ cells. Previous studies have showed, mice and human BMMSCs and ESCs differentiation to male germ cell lineage by using RA enriched medium (6, 8-10, 36, 37). By our study, it was found that although ADMSCs were attained easier and also cultured and differentiated rapidly, germ cell markers were expressed in BMMSCs significantly more than ADMSCs. 

Gamete production exclusively from stem cells not only can improve researchers’ conceptions of germ cells evolution, but also its benefits for fertility improvement can overcomes to ethical and technical criticisms. To understand if the germ cells derived from BMMSCs and ADMSCs can accomplish evolutionary process to achieve fertility or not, more studies are recommended regarding transferring the obtained germ cells to an infertile adult mouse testis. Also other studies can be designed to identify the effect of testicular extraction on differentiation process.

## Conclusion

In our study, BMMSCs and ADMSCs were cultured in appropriate media and differentiated to osteoblast and lipoblast that could express superficial mesenchymal characteristics. Differentiation to germ cells was induced by adding differentiating factors as essential supplements to the media. This experiment manifested that pluripotent ADMSCs and BMMSCs were capable to differentiate to germ cells. Although, ADMSCs were extracted more easily without painful complications and also rapid replication and differentiation, the superficial characteristics derived from these cells were expressed significantly less than the cells derived from BMMSCs
